# Supplementary material for: Effects of genomic location on ectopic integration and gene expression of a reporter gene cassette in Sulfolobus acidocaldarius
Source: Front Microbiol. 2025 Aug 5;16:1602937. doi: 10.3389/fmicb.2025.1602937 (PMC12361140; doi:10.3389/fmicb.2025.1602937)
Supplement: Supplementary file 1 [file Data_Sheet_1.pdf]

Supplementary Information

Effects of genomic location on ectopic integration and gene expression of a reporter gene cassette in *Sulfolobus acidocaldarius*

Yifei Xu[1], Andries Peeters[2], Indra Bervoets[1], Marjan De Mey[2], Rani Baes[1], Eveline Peeters[1]

[1]Research Group of Microbiology, Department of Bioengineering Sciences, Vrije Universiteit Brussel, Pleinlaan 2, B-1050 Brussels, Belgium.

[2]Centre for Synthetic Biology, Department of Biotechnology, Ghent University, Coupure links 653, B-9000 Ghent, Belgium.

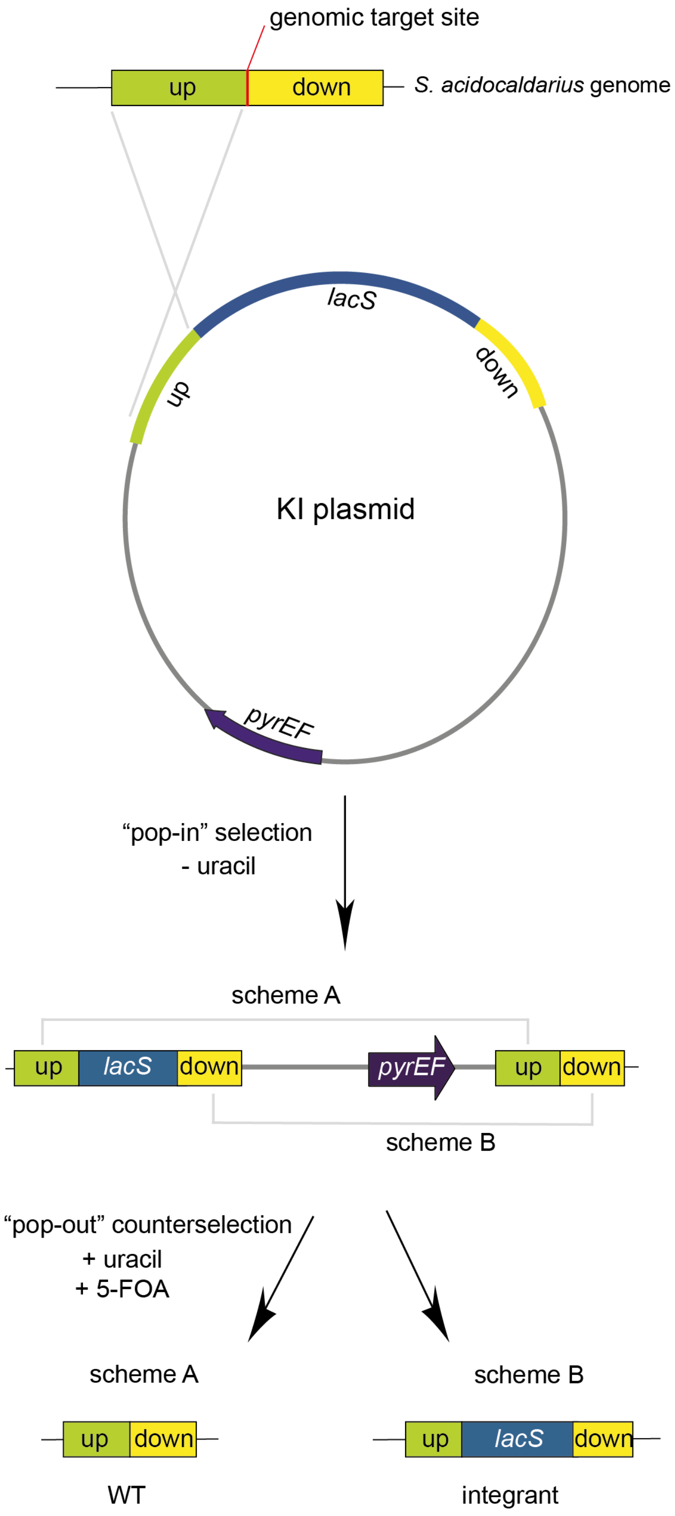

Supplementary Figure S1. Flowchart of the "pop-in pop-out" approach used for the construction of knock-in mutant strains in *S. acidocaldarius* SK-1. The genomic target site is indicated with a red line.

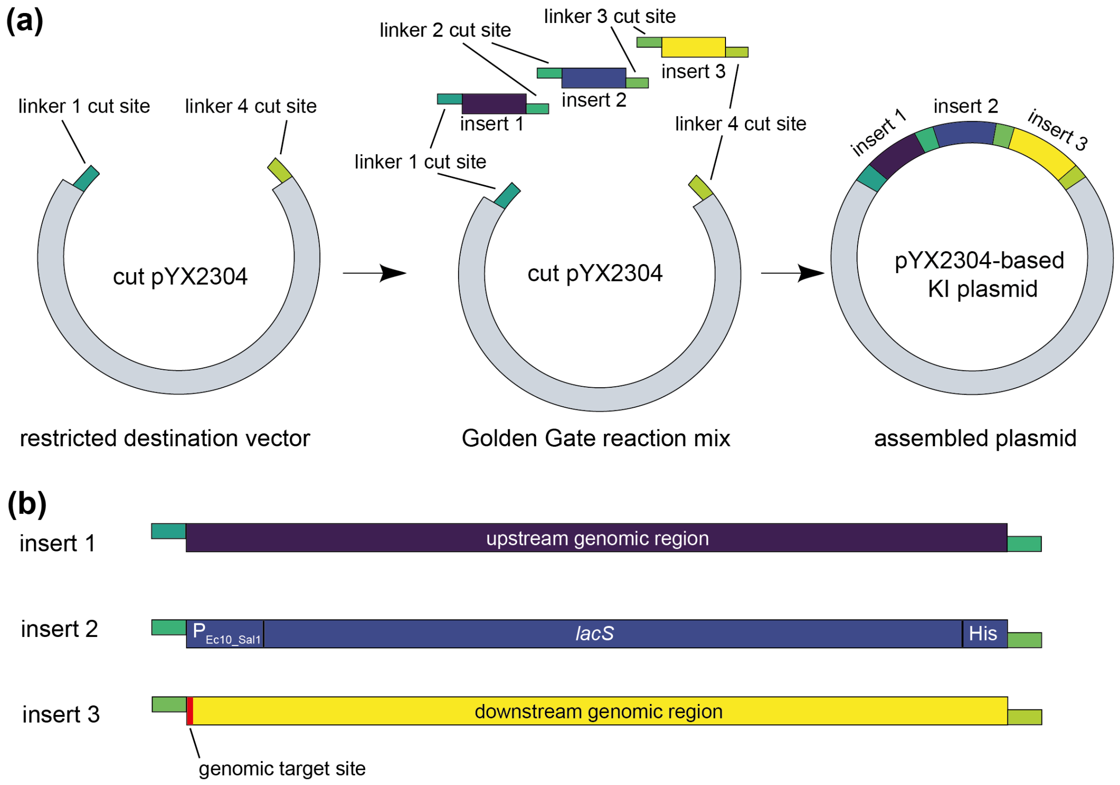

Supplementary Figure S2. Conceptual overview of the Golden Gate cloning scheme for the construction of knock-in (KI) plasmids. (**a**) Scheme of different steps in the cloning procedure with color-coded indication of different inserts and linker cut sites. The following type IIS restriction sites have been used as cut sites: linker 1 = 5’-AGGA-3’, linker 2 = 5’-TGAT-3’, linker 3 = 5’-GATG-3’ and linker 4 = 5’-GCAG-3’. More detailed plasmid maps are presented in **Figure 1**. (**b**) Identity of the different inserts, with indication of the P_{Ec10_Sal1} promoter and His-tag sequence in the *lacS* cassette (insert 2) and of the genomic target site in insert 3.

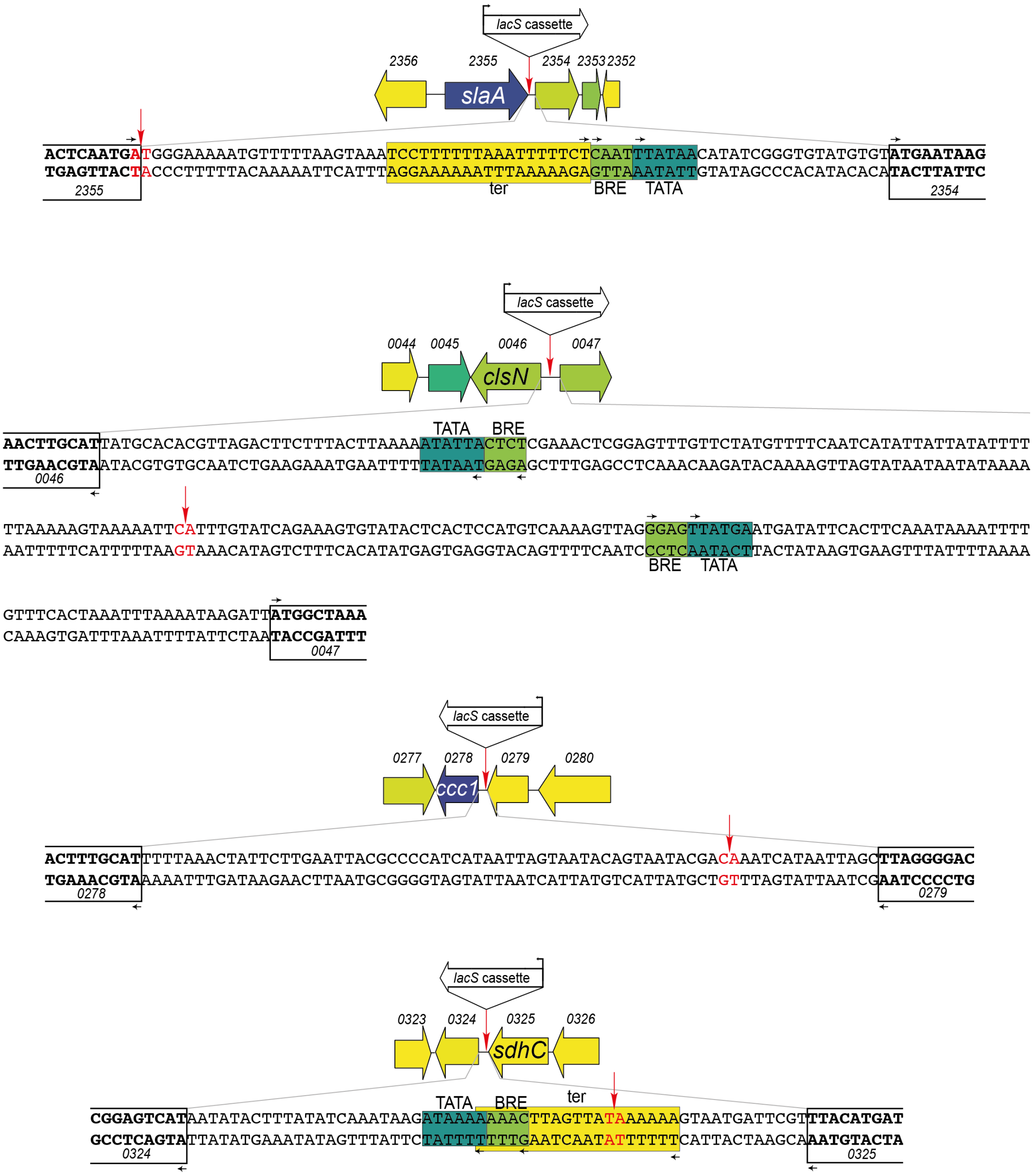

Supplementary Figure S3. Sequences of the genomic environment for each of the target sites for integration of the *lacS* cassette. The schematic depiction displays the target site and the orientation of the *lacS* cassette with respect to the genomic environment. Numbers refer to gene locus tags, with xxxx referring to *Saci_xxxx*. Gene arrows are color-coded according to expression levels (CPM value) (Baes et al. 2023) according to the color scheme shown in **Figure 3**. On the sequences, the target site is indicated in red, ORFs are boxed with the direction of transcription indicated by an arrow. Putative promoter elements are indicated with TATA = TATA box and BRE = factor B recognition element and putative terminator elements are indicated by ter. Arrows indicate the direction of transcription of the corresponding transcription unit.

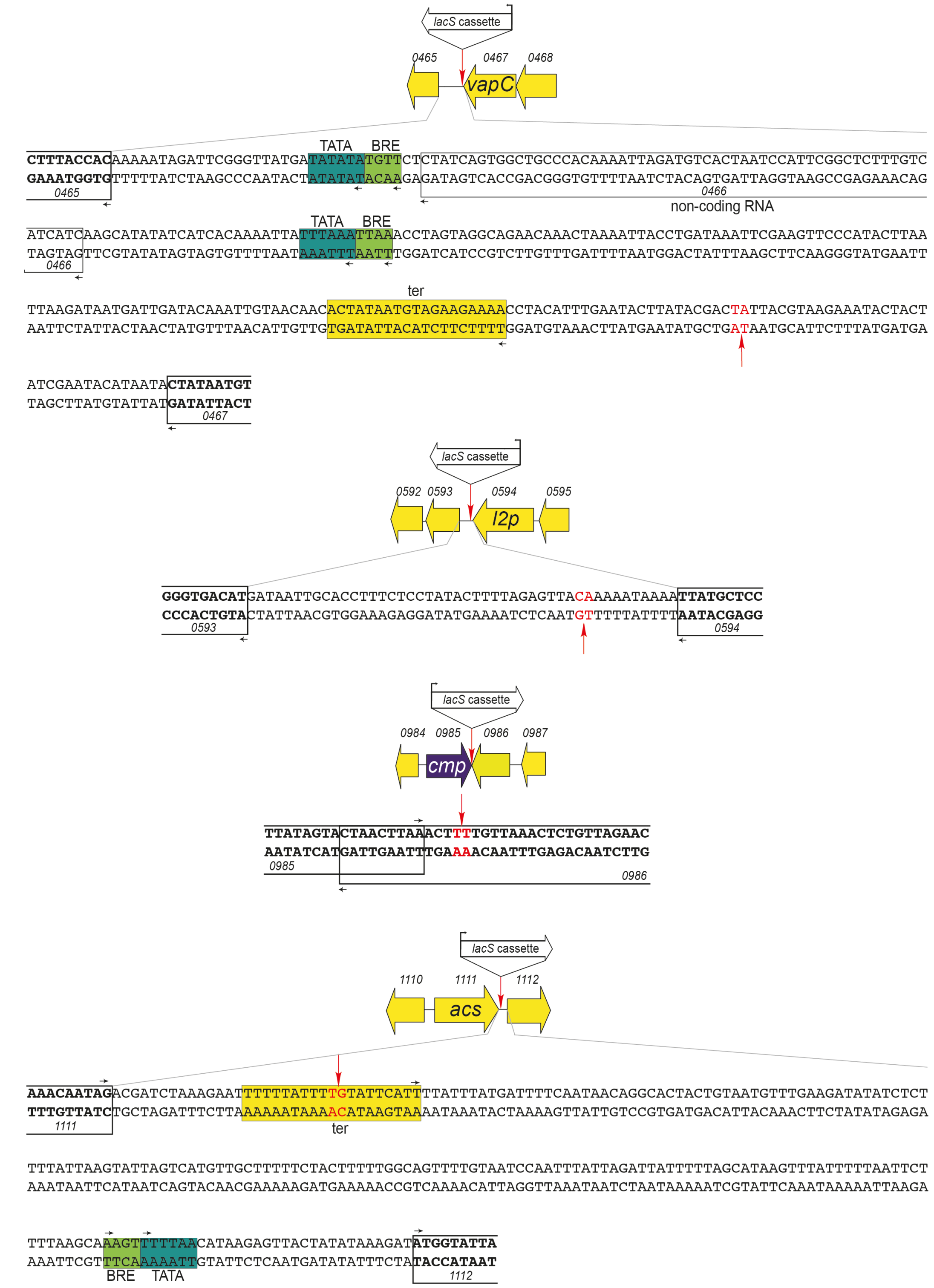

Supplementary Figure S3. *Continued.*

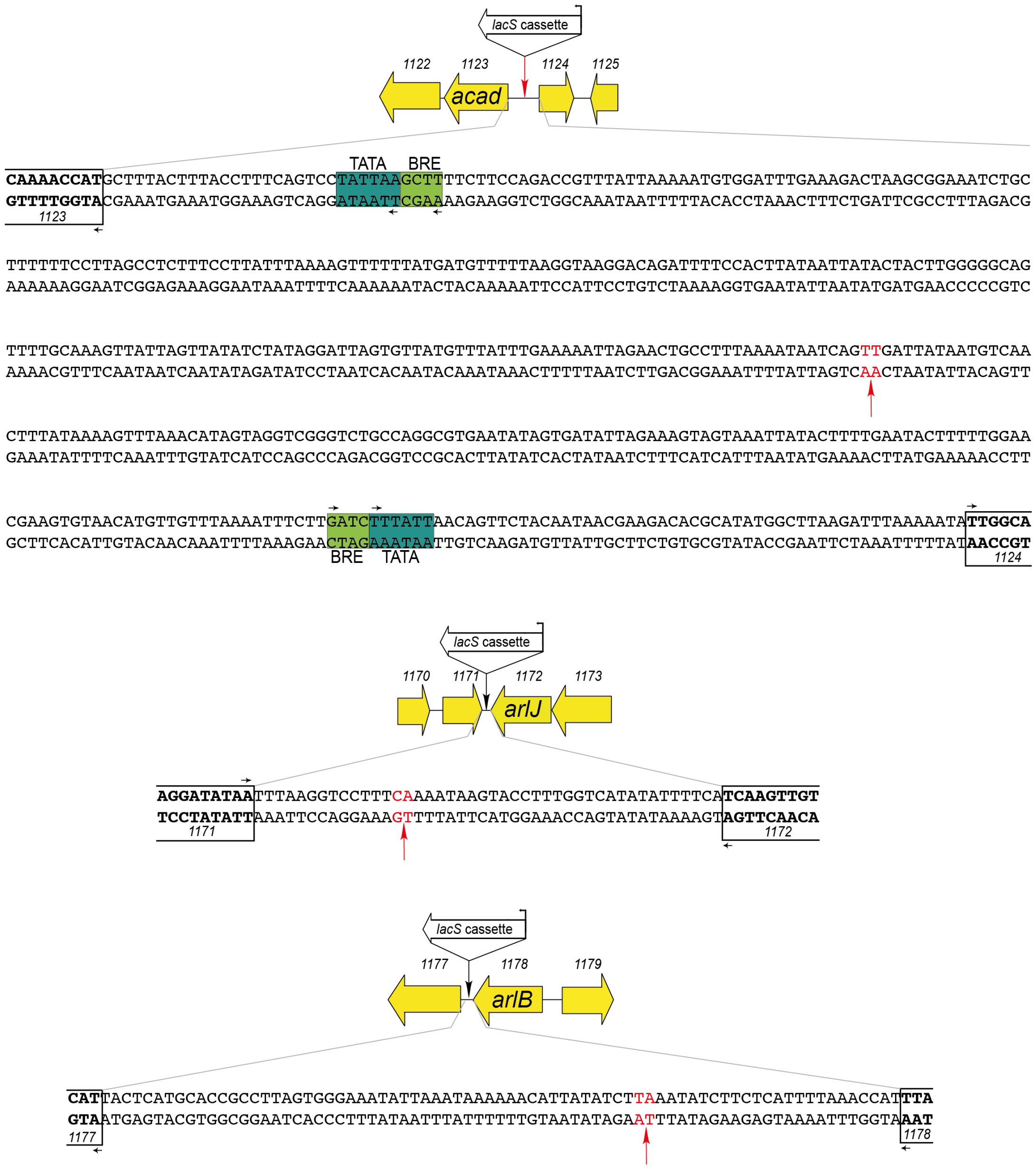

Supplementary Figure S3. *Continued.*

(a)

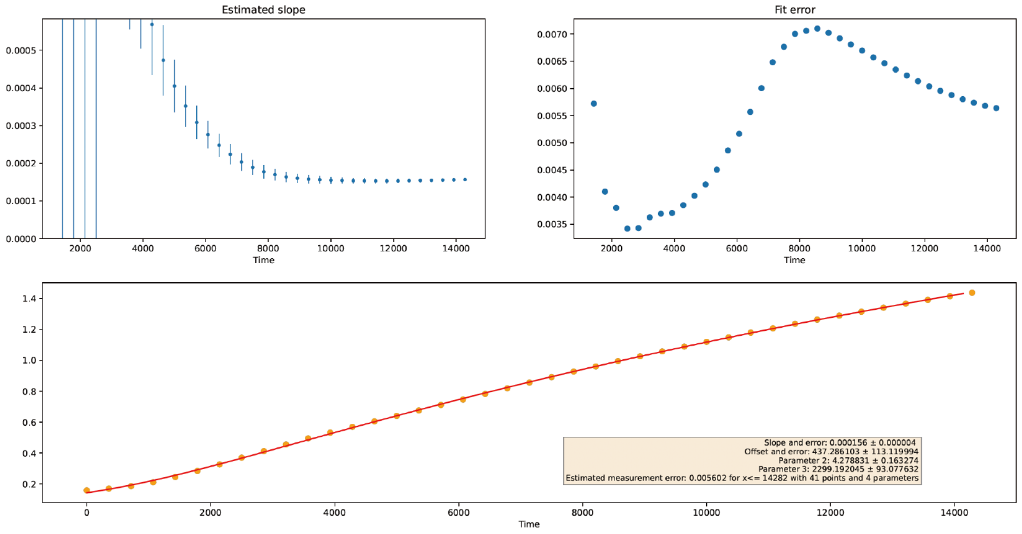

(b)

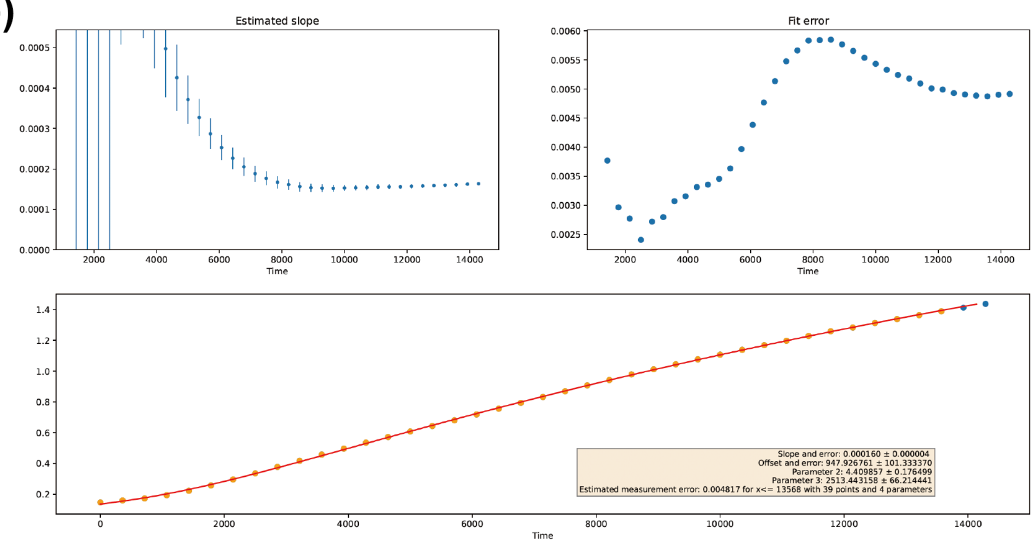

(c)

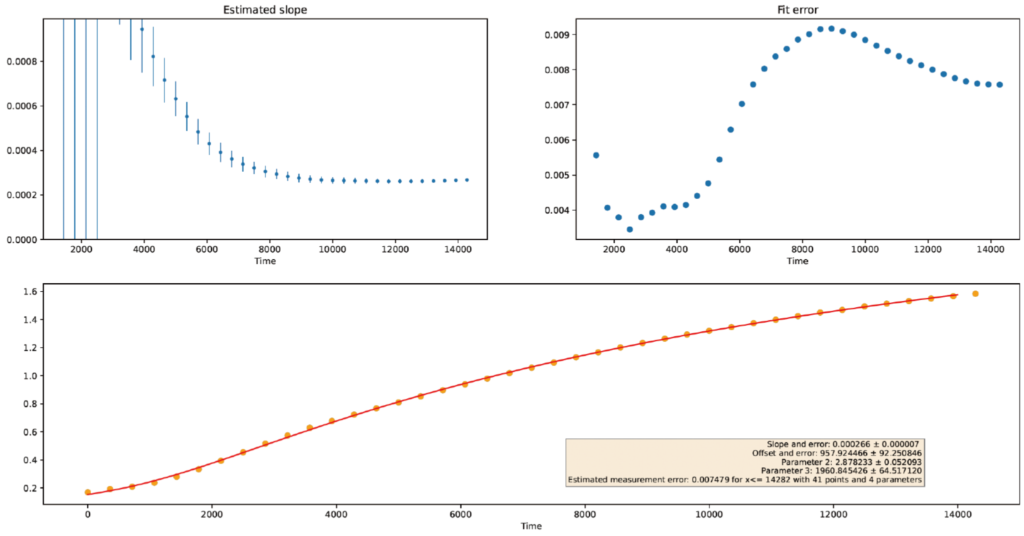

Supplementary Figure S4. Raw LacS activity data and its analysis for the first biological replicate of the slaA knock-in strain. For each technical replicate (**a**, **b** and **c**), the following information is shown: estimated slopes (top left), fit errors (top right) and absorbance at 420 nm (bottom). Parameter 2: m (maximum value) and parameter 3: d (period around the offset in which the transition takes place).

Supplementary Table S1. Overview of all microbial strains used in this work.

Name	Description	Origin
Escherichia coli DH5α	Host strain for cloning and plasmid propagation	Life Technologies
Sulfolobus acidocaldarius SK-1	Uracil-auxotrophic host strain	(Suzuki and Kurosawa 2016)
S. acidocaldarius SK1-*slaAx*KI*lacs*	Knock-in strain with a *lacS* cassette in the *slaA* locus	This study
S. acidocaldarius SK1-*clsNx*KI*lacs*	Knock-in strain with a *lacS* cassette in the *clsN* locus	This study
S. acidocaldarius SK1-*l2px*KI*lacs*	Knock-in strain with a *lacS* cassette in the *l2p* locus	This study
S. acidocaldarius SK1-*sdhCx*KI*lacs*	Knock-in strain with a *lacS* cassette in the *sdhC* locus	This study
S. acidocaldarius SK1-*fad11x*KI*lacs*	Knock-in strain with a *lacS* cassette in the *fad11* locus	This study
S. acidocaldarius SK1-*fad23x*KI*lacs*	Knock-in strain with a *lacS* cassette in the *fad23* locus	This study
S. acidocaldarius SK1-*flaBx*KI*lacs*	Knock-in strain with a *lacS* cassette in the *flaB* locus	This study
S. acidocaldarius SK1-*flaJx*KI*lacs*	Knock-in strain with a *lacS* cassette in the *flaJ* locus	This study
S. acidocaldarius SK1-*vapCx*KI*lacs*	Knock-in strain with a *lacS* cassette in the *vapC* locus	This study
S. acidocaldarius SK1-*cmpx*KI*lacs*	Knock-in strain with a *lacS* cassette in the *cmp* locus	This study
S. acidocaldarius SK1-*ccc1x*KI*lacs*	Knock-in strain with a *lacS* cassette in the *ccc1* locus	This study

Supplementary Table S2. Overview of oligonucleotides used in this work, with indication of their sequence and purpose. Golden Gate linker sequences are depicted in bold, with linker 1 = cut site 5'-AGGA-3', linker 2 = cut site 5'-TGAT-3', linker 3 = cut site 5'-GATG-3' and linker 4 = cut site 5'-GCAG-3'. For primer YX091, the sequence of the P_{Ec10_Sal1} promoter is indicated in bold.

Name	Sequence (5' – 3')	Purpose
YX091	**TATAATACAAATCACTCTCAAATGGTTTTATAAACTGAGGGGAGAGAATATTACTT**ATGGACTCATTTCCAAATAGCTTTAGGTTTG	Switch in pSVA431 from P*malE* to SP1 promoter.
YX123	CACCTGCCGATGCAGAATTCGCCCTATAGTGAGTCGTATTACAAT	Used with YX124 to amplify the backbone of pSVA431.
YX124	CACCTGCCGATTCCTGATAAGCATGCATGACCGGCTATT	Used with YX123 to amplify the backbone of pSVA431.
YX125	TCATGCATGCTTATCAGGAATCGGCAGGTGC	Used with YX126 to amplify the *sacB* gene.
YX126	ACTATAGGGCGAATTCTGCATCGGCAGGTGATGGGAT	Used with YX127 to amplify the *sacB* gene.
YX127	ATAAGGTGATGAAATGTAAAGGAGC	Used with YX128 for performing colony PCR and sequencing inserts in pYX2304.
YX128	GTAAAACGACGGCCAGT	Used with YX127 for performing colony PCR and sequencing inserts in pYX2304.
YX131	TATTCACCTGCACTA**AGGA**AGACTTCATTTGCAGTTTACACCAAT	Used with YX132 to amplify the upstream region of *slaA* locus, to assemble pYX2304-1. It contains a linker 1 for Golden Gate cloning.
YX132	TATTCACCTGCACTA**ATCA**TTGAGTTAATGTTACGTTTGTGAATATTAATTGT	Used with YX131 to amplify the upstream region of *slaA* locus, to assemble pYX2304-1. It contains a linker 2 for Golden Gate cloning.
YX133	TATTCACCTGCACTA**TGAT**ATAATACAAATCACTCTCAAATGGTTTTATAAACTGAGGG	Used with YX134 to amplify the *lacS* cassette, to assemble all pYX2304-based plasmids. It contains a linker 2 for Golden Gate cloning.
YX134	TATTCACCTGCACTA**CATC**AGTGGTGGTGGTGGTGGTGGTGCCTTAATGGCTTTACTGGA	Used with YX133 to amplify the *lacS* cassette, to assemble all pYX2304-based plasmids. It contains a linker 3 for Golden Gate cloning.
YX135	TATTCACCTGCACTA**GATG**GGAAAAAATGTTTTTAAGTAAATCCTTTTTTAAATTTTTCTC	Used with YX136 to amplify the downstream region of *slaA* locus, to assemble pYX2304-1. It contains a linker 3 for Golden Gate cloning.
YX136	TATTCACCTGCACTA**CTGC**CTTGAAGCGAAGCTACTAACTTAAATGT	Used with YX135 to amplify the downstream region of *slaA* locus, to assemble pYX2304-1. It contains a linker 4 for Golden Gate cloning.
YX137	AATTAGTGAAAAAATAGCCGGTC	Used with YX138 for sequencing of all pYX2304-based plasmid constructs.
YX138	GTAAAACGACGGCCAGT	Used with YX137 for sequencing of all pYX2304-based plasmid constructs.
YX139	TATAATACAAATCACTCTCAAATGG	Used with YX140 for sequencing of all pYX2304-based plasmid constructs.
YX140	GTGCCTTAATGGCTTTACTG	Used with YX139 for sequencing of all pYX2304-based plasmid constructs.
YX141	TATTCACCTGCACTA**AGGA**CTTTTCCTTTTCGAGCCTTTCAATTTCCA	Used with YX142 to amplify the upstream region of *clsN* locus, to assemble pYX2304-2. It contains a linker 1 for Golden Gate cloning.

YX142	TATTCACCTGCACTA**ATCA**GAATTTTTAC TTTTTAAAAAATATAATAATATGATTGAA AACATAGAACA	Used with YX141 to amplify the upstream region of *clsN* locus, to assemble pYX2304-2. It contains a linker 2 for Golden Gate cloning.
YX143	TATTCACCTGCACTA**GATG**ATTTGTATCA GAAAGTGTATACTCACTCCATGT	Used with YX144 to amplify the downstream region of *clsN* locus, to assemble pYX2304-2. It contains a linker 3 for Golden Gate cloning.
YX144	TATTCACCTGCACTA**CTGC**ATCAAACCCA GCTGAAACTACGATATTT	Used with YX143 to amplify the downstream region of *clsN* locus, to assemble pYX2304-2. It contains a linker 4 for Golden Gate cloning.
YX145	TATTCACCTGCACTA**AGGA**GCCAGCTAGA TATCCAAATATAGAGGGAGA	Used with YX146 to amplify the upstream region of *l2p* locus, to assemble pYX2304-3. It contains a linker 1 for Golden Gate cloning.
YX146	TATTCACCTGCACTA**ATCA**AAAAATAAAA TTATGCTCCACCACGTCTACC	Used with YX145 to amplify the upstream region of *l2p* locus, to assemble pYX2304-3. It contains a linker 2 for Golden Gate cloning.
YX147	TATTCACCTGCACTA**GATG**GTAACTCTAA AAGTATAGGAGAAAGGTGCAATTATCA	Used with YX148 to amplify the downstream region of *l2p* locus, to assemble pYX2304-3. It contains a linker 3 for Golden Gate cloning.
YX148	TATTCACCTGCACTA**CTGC**CAGAACGGTA AAGCTTCCTT	Used with YX147 to amplify the downstream region of *l2p* locus, to assemble pYX2304-3. It contains a linker 4 for Golden Gate cloning.
YX149	TATTCACCTGCACTA**AGGA**AATACTACAT GTCAAAATATCCTAAGGTCAGAGAGA	Used with YX150 to amplify the upstream region of *sdhC* locus, to assemble pYX2304-4. It contains a linker 1 for Golden Gate cloning.
YX150	TATTCACCTGCACTA**ATCA**AAAAAAGTAA TGATTCGTTTACATGTATCTCTTTAACAC TG	Used with YX149 to amplify the upstream region of *sdhC* locus, to assemble pYX2304-4. It contains a linker 2 for Golden Gate cloning.
YX151	TATTCACCTGCACTA**GATG**ATAACTTAAG TTTTTTTATCTTATTTGATATAAAGTATA TTATGACTCCG	Used with YX152 to amplify the downstream region of *sdhC* locus, to assemble pYX2304-4. It contains a linker 3 for Golden Gate cloning.
YX152	TATTCACCTGCACTA**CTGC**AATGATACTC ATAGAGGTACTAAAGAGTAATGCAGGAAC	Used with YX151 to amplify the downstream region of *sdhC* locus, to assemble pYX2304-4. It contains a linker 4 for Golden Gate cloning.
YX153	TATTCACCTGCACTA**AGGA**CTAAGCCCGA ATTCGAGCA	Used with YX154 to amplify the upstream region of *fad11* locus, to assemble pYX2304-5. It contains a linker 1 for Golden Gate cloning.
YX154	TATTCACCTGCACTA**ATCA**AAAATAAAAA ATTCTTTAGATCGTCTATTGTTTCATG	Used with YX153 to amplify the upstream region of *fad11* locus, to assemble pYX2304-5. It contains a linker 2 for Golden Gate cloning.
YX155	TATTCACCTGCACTA**GATG**GTATTCATTT TATTTATGATTTTCAATAACAGGCAC	Used with YX156 to amplify the downstream region of *fad11* locus, to assemble pYX2304-5. It contains a linker 3 for Golden Gate cloning.
YX156	TATTCACCTGCACTA**CTGC**TGCCTTTGGG AGCTCCT	Used with YX155 to amplify the downstream region of *fad11* locus, to assemble pYX2304-5. It contains a linker 4 for Golden Gate cloning.
YX157	TATTCACCTGCACTA**AGGA**GATAGTCAGT GAACCTTTCCG	Used with YX158 to amplify the upstream region of *fad23* locus, to assemble pYX2304-6. It contains a linker 1 for Golden Gate cloning.
YX158	TATTCACCTGCACTA**ATCA**TGATTATAAT GTCAACTTTATAAAAGTTTAAACATAGTA GG	Used with YX157 to amplify the upstream region of *fad23* locus, to assemble pYX2304-6. It contains a linker 2 for Golden Gate cloning.

YX159	TATTCACCTGCACTA**GATG**ACTGATTATTTTAAAGGCAGTTCTAATTTTTCA	Used with YX160 to amplify the downstream region of *fad23* locus, to assemble pYX2304-6. It contains a linker 3 for Golden Gate cloning.
YX160	TATTCACCTGCACTA**CTGC**CTTATCTCCCTTGGCAACAG	Used with YX159 to amplify the downstream region of *fad23* locus, to assemble pYX2304-6. It contains a linker 4 for Golden Gate cloning.
YX161	TATTCACCTGCACTA**AGGA**TATCTCCTTCAACTACAGCAATATCG	Used with YX162 to amplify the upstream region of *flaB* locus, to assemble pYX2304-7. It contains a linker 1 for Golden Gate cloning.
YX162	TATTCACCTGCACTA**ATCA**AAATATCTTCTCATTTTAAACCATTTATCCTATAACTG	Used with YX161 to amplify the upstream region of *flaB* locus, to assemble pYX2304-7. It contains a linker 2 for Golden Gate cloning.
YX163	TATTCACCTGCACTA**GATG**AAGATATAATGTTTTTTATTTAATATTTCCCACTAAGGC	Used with YX164 to amplify the downstream region of *flaB* locus, to assemble pYX2304-7. It contains a linker 3 for Golden Gate cloning.
YX164	TATTCACCTGCACTA**CTGC**GTAAACATATCATCTGAAGAGACATATCCTAATTC	Used with YX163 to amplify the downstream region of *flaB* locus, to assemble pYX2304-7. It contains a linker 4 for Golden Gate cloning.
YX165	TATTCACCTGCACTA**AGGA**TAAGGGGTGATTTAGGTTCTGC	Used with YX166 to amplify the upstream region of *flaJ* locus, to assemble pYX2304-8. It contains a linker 1 for Golden Gate cloning.
YX166	TATTCACCTGCACTA**ATCA**AAAATAAGTACCTTTGGTCATATATTTTCATCAAG	Used with YX165 to amplify the upstream region of *flaJ* locus, to assemble pYX2304-8. It contains a linker 2 for Golden Gate cloning.
YX167	TATTCACCTGCACTA**GATG**GAAAGGACCTTAAATTATATCCTGTTAAATATTTCT	Used with YX168 to amplify the downstream region of *flaJ* locus, to assemble pYX2304-8. It contains a linker 3 for Golden Gate cloning.
YX168	TATTCACCTGCACTA**CTGC**GCAATAAGAAAACTAGTTGACAAAGAAC	Used with YX167 to amplify the downstream region of *flaJ* locus, to assemble pYX2304-8. It contains a linker 4 for Golden Gate cloning.
YX169	TATTCACCTGCACTA**AGGA**TAAATATATTTATGCCTAAAGTCATTACAATATCAGATG	Used with YX170 to amplify the upstream region of *vapC* locus, to assemble pYX2304-9. It contains a linker 1 for Golden Gate cloning.
YX170	TATTCACCTGCACTA**ATCA**ATTACGTAAGAAATACTACTATCGAATACATAATACTATAAT	Used with YX169 to amplify the upstream region of *vapC* locus, to assemble pYX2304-9. It contains a linker 2 for Golden Gate cloning.
YX171	TATTCACCTGCACTA**GATG**AGTCGTATAAGTATTCAAATGTAGGTTTTCT	Used with YX172 to amplify the downstream region of *vapC* locus, to assemble pYX2304-9. It contains a linker 3 for Golden Gate cloning.
YX172	TATTCACCTGCACTA**CTGC**TTAGTATCCTCGAGTAGTTAAGAATCGAC	Used with YX171 to amplify the downstream region of *vapC* locus, to assemble pYX2304-9. It contains a linker 4 for Golden Gate cloning.
YX173	TATTCACCTGCACTA**AGGA**GTTATGGGAGAACTCAGTAAAGTATAATG	Used with YX174 to amplify the upstream region of *cmp* locus, to assemble pYX2304-10. It contains a linker 1 for Golden Gate cloning.
YX174	TATTCACCTGCACTA**ATCA**AAGTTTAAGTTAGTACTATAAAAATAATTGCAAGAAATAG	Used with YX173 to amplify the upstream region of *cmp* locus, to assemble pYX2304-10. It contains a linker 2 for Golden Gate cloning.
YX175	TATTCACCTGCACTA**GATG**TTGTTAAACTCTGTTAGAACATTGTCAACTT	Used with YX176 to amplify the downstream region of *cmp* locus, to assemble pYX2304-10. It contains a linker 3 for Golden Gate cloning.

YX176	TATTCACCTGCACTA**CTGC**CAGGGCGGTATGGGAT	Used with YX175 to amplify the downstream region of *cmp* locus, to assemble pYX2304-10. It contains a linker 4 for Golden Gate cloning.
YX177	TATTCACCTGCACTA**AGGA**TACATTTAACTTGGAGTTGTTACGAGG	Used with YX178 to amplify the upstream region of *ccc1* locus, to assemble pYX2304-11. It contains a linker 1 for Golden Gate cloning.
YX178	TATTCACCTGCACTA**ATCA**AAATCATAATTAGCTTAGGGGACAGC	Used with YX177 to amplify the upstream region of *ccc1* locus, to assemble pYX2304-11. It contains a linker 2 for Golden Gate cloning.
YX179	TATTCACCTGCACTA**GATG**GTCGTATTACTGTATTACTAATTATGATGGG	Used with YX180 to amplify the downstream region of *ccc1* locus, to assemble pYX2304-11. It contains a linker 3 for Golden Gate cloning.
YX180	TATTCACCTGCACTA**CTGC**TAACAAGTATAACAGCCATTGAGGAC	Used with YX179 to amplify the downstream region of *ccc1* locus, to assemble pYX2304-11. It contains a linker 4 for Golden Gate cloning.
YX183	TTAAGCGCAGTACCATTTGC	Used with YX184 for colony PCR of *S. acidocaldarius* SK1-*slaAx*KI*lacs*.
YX184	GCTAAGTTCTTATTCATACACATACAC	Used with YX183 for colony PCR of *S. acidocaldarius* SK1-*slaAx*KI*lacs*.
YX185	TCTAGCGATAATACTAGCTCTC	Used with YX186 for colony PCR of *S. acidocaldarius* SK1-*clsNx*KI*lacs*.
YX186	GACATGGAGTGAGTATACAC	Used with YX185 for colony PCR of *S. acidocaldarius* SK1-*clsNx*KI*lacs*.
YX187	GTCATATTGCATCAAGAAGGAC	Used with YX188 for colony PCR of *S. acidocaldarius* SK1-*l2px*KI*lacs*.
YX188	GATAATTGCACCTTTCTCCTA	Used with YX187 for colony PCR of *S. acidocaldarius* SK1-*l2px*KI*lacs*.
YX189	CCTATGTCAGTGTTAAAGAGATAC	Used with YX190 for colony PCR of *S. acidocaldarius* SK1-*sdhCx*KI*lacs*.
YX190	GTTAATTCCACCTCAGTATACG	Used with YX189 for colony PCR of *S. acidocaldarius* SK1-*sdhCx*KI*lacs*.
YX191	GGAGTTGAGAAATAAGTATAAGGAC	Used with YX192 for colony PCR of *S. acidocaldarius* SK1-*fad11x*KI*lacs*.
YX192	GTAGTGCCTGTTATTGAAAATC	Used with YX191 for colony PCR of *S. acidocaldarius* SK1-*fad11x*KI*lacs*.
YX193	ACAACATGTTACACTTCGTATC	Used with YX194 for colony PCR of *S. acidocaldarius* SK1-*fad23x*KI*lacs*.
YX194	CGGAAATCTGCTTTTTTTCC	Used with YX193 for colony PCR of *S. acidocaldarius* SK1-*fad23x*KI*lacs*.
YX195	CAATATCCCAGTATATCTATCAGC	Used with YX196 for colony PCR of *S. acidocaldarius* SK1-*flaBx*KI*lacs*.
YX196	CTTGGATAGCCATTACTCAT	Used with YX195 for colony PCR of *S. acidocaldarius* SK1-*flaBx*KI*lacs*.
YX197	CTGGAGGTATAATTAGTGGC	Used with YX198 for colony PCR of *S. acidocaldarius* SK1-*flaJx*KI*lacs*.
YX198	CGTTACTTTCGATATTCGCA	Used with YX197 for colony PCR of *S. acidocaldarius* SK1-*flaJx*KI*lacs*.
YX199	AAGTCTAATAATGAGGTTTTGATAAC	Used with YX200 for colony PCR of *S. acidocaldarius* SK1-*vapCx*KI*lacs*.
YX200	GATTGATACAAATTGTAACAACACT	Used with YX199 for colony PCR of *S. acidocaldarius* SK1-*vapCx*KI*lacs*.

YX201	GGAGGTTATGTTATGAAAACATTC	Used with YX202 for colony PCR of *S. acidocaldarius* SK1-*cmpx*KI*lacs*.
YX202	CTTTAGGTAGTTGGAGAAGAGA	Used with YX201 for colony PCR of *S. acidocaldarius* SK1-*cmpx*KI*lacs*.
YX203	AATGGGGTATATACTCCAATTTC	Used with YX204 for colony PCR of *S. acidocaldarius* SK1-*cc1x*KI*lacs*.
YX204	GAGTCCATCTTGTATTCCG	Used with YX203 for colony PCR of *S. acidocaldarius* SK1-*cc1x*KI*lacs*.

Supplementary Table S3. Overview of all plasmids used in this work.

Name	Description	Origin
pSVA431	Suicide vector for construction of mutant strains	(Wagner et al. 2012)
pYX2301-UPDD	pSVA431 variant with SP1 promoter	This study
pRN1-carrier_2_paqci	Construction of Golden Gate destination vector	This study
pYX2304	Golden Gate destination vector	This study
pYX2304-1	Suicide vector for construction of *S. acidocaldarius* SK1-*slaAx*KI*lacs*	This study
pYX2304-2	Suicide vector for construction of *S. acidocaldarius* SK1-*clsNx*KI*lacs*	This study
pYX2304-3	Suicide vector for construction of *S. acidocaldarius* SK1-*l2px*KI*lacs*	This study
pYX2304-4	Suicide vector for construction of *S. acidocaldarius* SK1-*sdhCx*KI*lacs*	This study
pYX2304-5	Suicide vector for construction of *S. acidocaldarius* SK1-*fad11Ax*KI*lacs*	This study
pYX2304-6	Suicide vector for construction of *S. acidocaldarius* SK1-*fad23x*KI*lacs*	This study
pYX2304-7	Suicide vector for construction of *S. acidocaldarius* SK1-*flaBx*KI*lacs*	This study
pYX2304-8	Suicide vector for construction of *S. acidocaldarius* SK1-*flaJx*KI*lacs*	This study
pYX2304-9	Suicide vector for construction of *S. acidocaldarius* SK1-*vapCx*KI*lacs*	This study
pYX2304-10	Suicide vector for construction of *S. acidocaldarius* SK1-*cmpx*KI*lacs*	This study
pYX2304-11	Suicide vector for construction of *S. acidocaldarius* SK1-*ccc1x*KI*lacs*	This study

Supplementary Table S4. Comparison between relative transcriptional expression level of *lacS* as measured by qRT-PCR in the different knock-in strains, relative to the *arlJ* knock-in strain (**Figure 5a**) and expression levels of the adjacent gene of which the transcription unit is most closely located to the *lacS* cassette, expressed in CPM value (Baes et al. 2023).

Knock-in strain	Log_2 fold ratio *lacS* expression	Adjacent gene of which the transcription unit is most closely located to the *lacS* cassette	Expression level
slaA	5.77	*saci_2355*	11554
ccc1	2.29	*saci_0279*	55012
sdhC	0.00	*saci_0325*	129.5
vapC	2.28	*saci_0467*	1.47
l2p	1.16	*saci_0594*	53.09
arlJ	0.00	*saci_1172*	257.89
arlB	0.62	*saci_1178*	35.18

Supplementary Table S5. Differential transcriptional expression of genes nearby integration target sites after 4 hours of nutritional starvation *versus* before nutritional starvation. Data have been extracted from https://www.ncbi.nlm.nih.gov/geo/query/acc.cgi?acc=GSE113716 (Bischof et al. 2018).

Gene	Relevant for knock-in strain:	Log_2 fold change	Padj
saci_0046	*clsN*	1.226436551	3.21e-05
saci_0047	*clsN*	1.45494138	0.018070789
saci_0278	*ccc1*	1.078734193	1.43e-08
saci_0279	*ccc1*	0.463199511	0.449680086
saci_0324	*sdhC*	-0.07497738	0.691914518
saci_0325	*sdhC*	1.132400907	0.00682609
saci_0465	*vapC*	-0.632702675	0.052983597
saci_0467	*vapC*	-0.162542615	0.163992396
saci_0593	*l2p*	-1.461100727	0.009841584
saci_0594	*l2p*	-1.680647198	0.019528253
saci_0985	*cmp*	-2.58973373	3.08e-19
saci_0986	*cmp*	0.364089912	0.283935228
saci_1111	*acs*	2.138177212	7.76e-12
saci_1112	*acs*	1.174701919	1.45e-10
saci_1123	*acad*	0.429453768	0.879249398
saci_1124	*acad*	1.970245477	0.01197094
saci_1171	*arlJ*	-2.110737372	3.1e-08
saci_1172	*arlJ*	1.469024275	3.69e-05
saci_1177	*arlB*	3.601347319	5.58e-25
saci_1178	*arlB*	4.385035176	3.11e-32
saci_2354	*slaA*	-0.289708086	0.918094922
saci_2355	*slaA*	-0.443885949	0.481090249

Supplementary Table S6. Differential transcriptional expression of genes nearby integration target sites in stationary *versus* exponential growth phase. Data have been extracted from https://www.ncbi.nlm.nih.gov/geo/query/acc.cgi?acc=GSE128063 (Takemata et al. 2019). Statistics are not included for these estimated $\log_2$ fold change values, which are based on TPM read files and should thus be interpreted with caution.

Gene	Relevant for knock-in strain:	Log_2 fold change
saci_0046	*clsN*	1.56
saci_0047	*clsN*	-2.30
saci_0278	*ccc1*	0.09
saci_0279	*ccc1*	-0.03
saci_0324	*sdhC*	-4.00
saci_0325	*sdhC*	2.09
saci_0465	*vapC*	-2.54
saci_0467	*vapC*	-1.36
saci_0593	*l2p*	1.59
saci_0594	*l2p*	1.55
saci_0985	*cmp*	-3.03
saci_0986	*cmp*	1.53
saci_1111	*acs*	-3.01
saci_1112	*acs*	-1.44
saci_1123	*acad*	-0.57
saci_1124	*acad*	-5.11
saci_1171	*arlJ*	-1.71
saci_1172	*arlJ*	-1.45
saci_1177	*arlB*	2.04
saci_1178	*arlB*	5.64
saci_2354	*slaA*	4.38
saci_2355	*slaA*	5.73

Supplementary references

Bischof, L.F., Haurat, M.F., Hoffman, L., Albersmeier, A., Wolf, J., Neu, A., Pham, T.K., Albaum, S.P., Jakobi, T., Schouten, S., Neumann-Schaal, M., Wright, P.C., Kalinowksi, J., Siebers, B. & Albers, S.-V. (2018). Early response of *Sulfolobus acidocaldarius* to nutrient limitation. Frontiers in Microbiology, 9, 3201.

Baes, R., Grünberger, F., Pyr Dit Ruys, S., Couturier, M., De Keulenaer, S., Skevin, S., Van Nieuwerburgh, F., Vertommen, D., Grohmann, D., Ferreira-Cerca, S., & Peeters, E. (2023). Transcriptional and translational dynamics underlying heat shock response in the thermophilic crenarchaeon *Sulfolobus acidocaldarius*. mBio, 14(5), e0359322.

Suzuki, S., & Kurosawa, N. (2016). Disruption of the gene encoding restriction endonuclease SuaI and development of a host-vector system for the thermoacidophilic archaeon *Sulfolobus acidocaldarius*. Extremophiles, 20(2), 139–148.

Takemata, N., Samson, R. Y., & Bell, S. D. (2019). Physical and Functional Compartmentalization of Archaeal Chromosomes. Cell, 179(1), 165-179.

Wagner, M., M. van Wolferen, A. Wagner, K. Lassak, B. H. Meyer, J. Reimann and S.-V. Albers (2012). Versatile Genetic Tool Box for the Crenarchaeote *Sulfolobus acidocaldarius*. Frontiers in Microbiology, 3, 214.
